# Prevalence of Oral Habits in Children with Cleft Lip and Palate

**DOI:** 10.1155/2013/247908

**Published:** 2013-02-27

**Authors:** Paula Caroline Barsi, Thaieny Ribeiro da Silva, Beatriz Costa, Gisele da Silva Dalben

**Affiliations:** Pediatric Dentistry, Hospital for Rehabilitation of Craniofacial Anomalies, University of São Paulo (HRAC/USP), R. Silvio Marchione, 3-20-Vila Universitária, 17012-900 Bauru, SP, Brazil

## Abstract

This study investigated the prevalence of oral habits in children with clefts aged three to six years, compared to a control group of children without clefts in the same age range, and compared the oral habits between children with clefts with and without palatal fistulae. The sample was composed of 110 children aged 3 to 6 years with complete unilateral cleft lip and palate and 110 children without alterations. The prevalence of oral habits and the correlation between habits and presence of fistulae (for children with clefts) were analyzed by questionnaires applied to the children caretakers. The cleft influenced the prevalence of oral habits, with lower prevalence of pacifier sucking for children with cleft lip and palate and higher prevalence for all other habits, with significant association (*P* < 0.05). There was no significant association between oral habits and presence of fistulae (*P* > 0.05). The lower prevalence of pacifier sucking and higher prevalence of other oral habits agreed with the postoperative counseling to remove the pacifier sucking habit when the child is submitted to palatoplasty, possibly representing a substitution of habits. There was no causal relationship between habits and presence of palatal fistulae.

## 1. Introduction

The manner how children are raised is very important for their full development, general health, and inclusion or exclusion of costumes and habits.

Habit is a behavior acquired by the frequent repetition or physiologic exposure with regularity [1]. Related to the mouth, it is commonly observed in children and may be harmful when excessively repeated or in more vulnerable ages. They often involve patterns of muscle contraction and may contribute to the etiology of malocclusion, because they affect the entire orofacial region.

In the presence of habits, the duration of the applied force is the most critical variable to be analyzed, because the longer the duration, the greater will be the impact on the dentition, musculature, and bone structure [2].

Considerable differences are observed in the prevalence of habits throughout the world. Traditions, cultural influences, and child raising are possible factors that influence their prevalence. The prevalence of sucking habits in Brazil seems to vary between states because of differences in culture, ethnicity, and lifestyle [3].

The period of breastfeeding has been indicated as a possible cause of nonnutritive sucking habits [3]. Holanda et al. [4] stated that breastfeeding for longer than six months is considered a protective factor against the persistence of pacifier use but highlighted that the affective relationship between mother and child during breastfeeding and after this period should be further investigated to better understand the etiology of nonnutritive sucking habits. The extended breastfeeding seems to have a healthy psychological impact and possibly provide a greater sensation of confidence and safety during child development. The higher income and educational level of parents are also associated with sucking habits, such as pacifier sucking at the age range from 3 to 5 years [4].

Nonnutritive sucking habits are risk factors for the occurrence of anterior open bite and posterior crossbite. Heimer et al. [5] observed a significant reduction in the prevalence of anterior open bite with age, suggesting the self-correction of this malocclusion when the habit is discontinued.

Anxiety, stress, and loneliness may also trigger habits as nail biting, commonly observed in children, which may be originated from the thumb-sucking habit that is transferred to the nails. The clinical examination of these patients reveals tooth crowding, rotation and wear of edges of mandibular incisors, and protrusion of maxillary incisors [6].

The recognition and elimination of oral habits are extremely important also for the prognosis of periodontal diseases. Some oral habits are considered cofactors in the development of gingival recessions [7].

The sucking process is observed early at 29 weeks of intrauterine life and is the first muscle coordination activity of the child [8]. Even though the sucking habit is very common during childhood and continued up to the second year of life, immediate intervention is necessary in children with operated clefts, because the habits have a great influence on the treatment outcome of cleft lip and palate. The pressure applied on the oral cavity muscles during sucking habits interferes with the repair of cleft lip and palate [8].

The literature on oral habits in children with cleft lip and palate is scarce. The objective of this study was to investigate the oral habits among individuals with operated cleft with and without palatal fistulae, compared to individuals without clefts.

## 2. Material and Methods

The project was approved by the Institutional Review Board (protocol number 286/2011). The study was conducted on 110 children with complete unilateral cleft lip and palate aged three to six years, with or without palatal fistulae, attending a reference craniofacial center in Brazil. Children were included regardless of ethnicity and gender. Children with associated anomalies, syndromes, and/or neuropsychomotor developmental disorders were excluded.

Data were collected by a questionnaire responded by the caretakers. Before onset, this questionnaire was applied to ten individuals (not participating in the study) to check if the caretakers might have any doubt in indicating their responses. The questionnaire consisted of a form indicating the several types of oral habits in which the caretakers had to choose between “yes” or “no” and indicate the duration and frequency of the habit (since when/how often).

These questionnaires were applied to caretakers of two groups of children. The first (study) group comprised children attending the pediatric dental clinic of the craniofacial center during the study period. This group was further divided in two subgroups, namely, with or without palatal fistulae. To evaluate the presence of these fistulae, children were submitted to clinical examination using a dental mirror and tongue depressor, under artificial light, by a single examiner. Palatal fistulae were considered as present regardless of their size and location, either in the hard, intermediate or soft palate.

The second (control) group was composed of children without clefts aged three to six years, attending a nursery center in the city of Bauru, for comparison of results between children with and without clefts.

The prevalence of oral habits between children with and without clefts was compared by the Fisher test. The presence of oral habits between children with clefts with or without fistulae was assessed by the Fisher test followed by the Chi-square test. All statistical tests were applied at a significance level of *P* < 0.05.

## 3. Results

All children with clefts had already been submitted to surgical repair. In this group, 65.5% of clefts affected the left side and fistulae were observed in 42.72%, primarily affecting the hard palate (74.5%), followed by the intermediate palate (17.1%), soft palate (4.2%), and intermediate and hard palate (4.2%). No significant association was observed between oral habits and the presence of palatal fistulae according to the Fisher test (*P* > 0.05).

**Figure 1:**
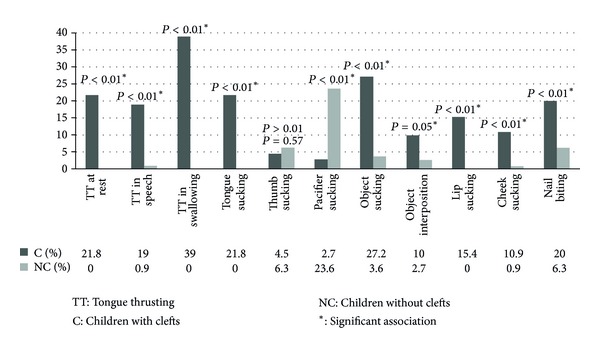
Prevalence of oral habits in children with cleft lip and palate compared to the control group.

The results for both groups are presented in Figure 1. When compared to the control group, children with clefts presented significant association with tongue thrusting at rest, in speech and in swallowing, tongue sucking, object sucking and interposition, lip sucking, cheek sucking, and nail biting. Conversely, there was significant association between the presence of cleft and lack of pacifier sucking habit. There was no significant association (*P* > 0.05) with thumb and finger sucking.

## 4. Discussion

This study analyzed the prevalence of oral habits in children with cleft lip and palate compared to children without clefts, correlating possible causes and interferences. Data were collected by a questionnaire applied to the caretakers and relied on their responses; thus, the following discussions should be considered under the light of caretakers' reports. Mainly, the findings revealed lower prevalence of pacifier sucking and higher prevalence of other habits in children with clefts compared to children without clefts.

Silva Filho et al. [9] analyzed the most common habits in children without clefts and reported pacifier sucking among the most frequent (28.95%). In the present study, the prevalence of pacifier sucking was higher in children without clefts compared to children with clefts. In general, the prevalence of sucking habits in children has been associated with several factors including age, gender, ethnicity, number of siblings, and socioeconomic status [10]. This study further suggests that lip and palate repair surgeries performed early may also interfere with the prevalence of oral habits as pacifier sucking.

Satyaprasad [8] reported that even though some oral habits are very common during childhood and persist up to the second year of life, immediate intervention is necessary in children with clefts because they may have a harmful influence on the treatment outcome of cleft lip and palate. The results of their electromyography study of several orofacial muscles revealed that they remain active during sucking habits, thus possibly altering the outcome of cleft treatment.

Patients submitted to surgeries for lip and palate repair also present alterations in muscle functions in the orofacial region. Due to the difference in the adaptability and functions of muscles in the postoperative period, interventions and additional care are necessary [8]. Therefore, the parents are commonly advised by medical doctors, especially plastic surgeons, to remove the pacifier sucking habit of their children after the repair surgeries.

Mothers routinely report that they do not even offer the pacifier to the child to avoid the establishment of the habit. Almeida et al. [11] reported that finger or pacifier sucking is normal in the onset of child development, and the opposition of parents to these habits may cause negative psychological consequences to the child. Franco et al. [12] suggests knowledge on the etiology of acquisition of sucking habits and how they may be harmful to allow their prevention by follow-up and counseling to the parents.

Holanda et al. [4] highlighted that the pacifier-sucking habit is significantly associated with age (3–5 years), with greater association at the age of 3 years. The present study included children aged 3 to 6 years to investigate the prevalence of oral habits and revealed that, in the case of children with clefts, pacifier sucking is interrupted early by the parents because of the lip and palate surgery, thus being uncommon in this group of children.

Franco et al. [12] confirmed that, in children with clefts, the acquisition of sucking habits may be influenced by the repair surgeries at early ages, because they use arm retainers in the first month after surgery, which precludes placement of the hand and objects in the mouth, to avoid trauma and infection. Interruption of pacifier sucking is also recommended, making children to abandon the sucking habit often present in earlier periods.

Considering the age range included in the study, all children in the sample had already been operated, since lip repair in the institution is usually performed at three months of age. The higher prevalence of other habits than pacifier sucking, such as tongue thrusting at rest, in speech and swallowing, tongue sucking, interposition and sucking of other objects, and lips, cheeks, and nail biting in the group of children with clefts probably represents a substitution of habits by the children, who are restrained from using the pacifier in the postoperative period. It should be highlighted that such other habits may also be harmful to the development of dental occlusion. Habits as tongue thrusting and sucking may be difficult to manage because of the prompt availability of the involved structure, that is, the tongue rather than a foreign object. Therefore, these children should be followed and their caretakers properly counseled concerning the possible occurrence of such habits to avoid their establishment or allow early intervention.

This study did not demonstrate correlation between oral habits and presence of fistulae. Passos et al. [13] conducted a study in the same institution as the present investigation and observed that 27% of subjects in their study presented palatal fistulae, reporting that the occurrence of palatal fistulae after primary palatoplasty is not uncommon.

Passos et al. [13] further reported that, after discharge, the caretakers of patients are advised by the nursing team and receive a handout with information on the postoperative care that must be followed until complete healing of the palate. However, doubts may arise on the compliance with this care and how this might significantly influence the formation of fistulae, considering that many individuals assisted at the institution present low socioeconomic cultural level, in addition to the overindulgence observed in families of children with clefts.

Investigation of the prevalence of oral habits and correlation between fistula and oral habits in children with cleft lip and palate is fundamental to allow better knowledge and confidence of professionals treating these patients, who may then offer better counseling for the patients' parents or caretakers.

In conclusion, considering the medical orientation on the need to remove the pacifier-sucking habit when the child is submitted to palatoplasty, due to the difference of adaptability and muscular functions in the postoperative period, the present findings reflect such advice, revealing lower prevalence of pacifier sucking and higher prevalence of other oral habits, supposedly a substitution of habits. No relationship was observed between habits and presence of palatal fistulae or dehiscences. Of course, the occurrence of palatal fistulae may also be influenced by other factors such as surgeon's skill, postoperative infection, and care, besides others [13]. However, the present findings suggest the need of a prospective, randomized study to assess the actual influence of oral habits on the postoperative outcome, considering the possibility of substitution by other habits and their long-term consequences in the children's lives.
